# Flavonoid Composition and Molecular Basis of the Potential Sexual-Enhancing Properties of a *Turnera diffusa* Extract (Liboost^®^)

**DOI:** 10.3390/ph19040597

**Published:** 2026-04-08

**Authors:** Iván Benito-Vázquez, María Inés Morán-Valero, Marina Díez-Municio, Adal Mena-García

**Affiliations:** 1 Instituto de Investigación en Ciencias de la Alimentación, CIAL (CSIC-UAM), Nicolás Cabrera 9, 28049 Madrid, Spain; ivan.benito@pharmactive.eu; 2 Pharmactive Biotech Products SLU, Faraday, 7, 28049 Madrid, Spain; ines.moran@pharmactive.eu (M.I.M.-V.); mdiez@pharmactive.eu (M.D.-M.)

**Keywords:** *Turnera diffusa*, flavonoids, phosphodiesterase-5 (PDE5), nitric oxide, aromatase inhibition

## Abstract

**Background/Objectives:** Sexual dysfunction is a prevalent and multifactorial condition affecting a large proportion of the global population, with limited therapeutic options beyond pharmacological approaches primarily targeting erectile dysfunction. This has increased interest in botanical supplements for sexual health, although mechanistic evidence and clear links between phytochemical composition and biological activity remain scarce. The present study provides an integrative evaluation of a commercial *Turnera diffusa* extract (Liboost^®^) formulated to support sexual health by combining detailed phytochemical characterization with targeted in vitro mechanistic assays. **Methods:** The extract was characterized by HPLC-DAD-HRMS, enabling the identification and semi-quantification of its major constituents. A total of 49 compounds were detected, predominantly flavonoids, including luteolin- and apigenin-derived glycosides, flavonols, methoxyflavones, flavanones, and coumaroyl derivatives, with a total quantified flavonoid content of 15.9 mg·g^−1^. Biological activity was evaluated in human cell models without cytotoxic effects at the tested concentrations. **Results:** Liboost^®^ significantly reduced PDE5 expression, inhibited aromatase activity, and moderately increased nitric oxide production. These complementary effects suggest a multi-target modulation of pathways involved in sexual function, integrating vascular, endocrine, and nitrergic mechanisms. **Conclusions:** Although limited to in vitro models, the findings provide mechanistic support for the biological activity of *T. diffusa* extracts and highlight the importance of linking phytochemical composition with functional evidence when evaluating botanical supplements.

## 1. Introduction

Sexual dysfunction represents a major global health issue, encompassing a spectrum of disorders that compromise sexual desire, arousal, and performance. It is a prevalent and multifactorial condition that affects approximately 30% of men and 43% of women worldwide. Although the incidence and prevalence of sexual dysfunction vary considerably across regions and populations, the most frequently reported conditions are erectile dysfunction in men and low sexual desire in women. Moreover, the prevalence of these disorders increases with age and is further exacerbated by comorbidities such as diabetes and cardiovascular disease [1,2,3,4].

Penile and female genital arousal rely on a coordinated neurovascular-endocrine mechanism dominated by nitric oxide (NO), cyclic guanosine monophosphate (cGMP), and sex steroid regulation. In men, erection is initiated by neuronal NO released from neural Nitric Oxide Synthase (nNOS)-positive autonomic fibers, whose essential role is demonstrated by the complete abolition of nerve-evoked erection after NOS inhibition [5]. Female genital tissue exhibits an analogous organization, since both nNOS and endothelial NOS (eNOS) are present in the human clitoris [6]. NO activates guanylate cyclase (GC) and increases cGMP in penile, clitoral and vaginal smooth muscle, producing acute vasodilation [7]. Sustained engorgement requires endothelial NO, as eNOS activity is tightly regulated by estrogen-dependent phosphorylation and caveolin-1 dynamics, decreasing substantially under estrogen-deficient conditions [8]. PDE5, expressed in clitoral and vaginal tissue, limits cGMP bioavailability, and its inhibition enhances genital relaxation in preclinical models and selected clinical cohorts [9,10]. Sexual desire, particularly in women, is strongly modulated by androgens, with total and free testosterone, androstenedione and DHEAS showing positive associations with desire and arousal [11]. Aromatase regulates this androgen–estrogen balance [12], and its inhibition increases systemic testosterone levels [12], while androgens facilitate NO-dependent vaginal smooth-muscle relaxation [8]. Together, these pathways define the biological basis underlying sexual desire, arousal and performance in both sexes.

Despite the high global burden of sexual dysfunction, current therapeutic strategies remain narrowly focused on a limited subset of conditions. Over the past two decades, pharmacological developments have primarily targeted the vascular mechanisms underlying erectile dysfunction, culminating in the widespread use of phosphodiesterase type 5 (PDE5) inhibitors [13]. However, the limited availability of effective treatments for other sexual disorders, together with the side effects associated with existing medications, has driven growing interest in herbal supplements as alternatives that are perceived safer and potentially effective [14,15].

This shift is reflected in the rapid expansion of the sexual health supplement market, which was valued at approximately USD 3.13 billion in 2025 and is projected to reach USD 4.95 billion by 2029, with a compound annual growth rate (CAGR) of 9.5% [16]. Nevertheless, despite the long-standing use of botanical preparations to enhance sexual function, the scientific evidence supporting most natural aphrodisiacs remains scarce, fragmented, or largely anecdotal [17,18,19,20]. Compounding this concern, sexual health supplements constitute the primary target for adulteration, accounting for more than 50% of fraudulent products on the US market, a trend that has continued to increase in recent years [21].

Within this growing landscape, particular attention has been directed toward botanicals traditionally associated with sexual health. Among these botanicals, damiana (*Turnera diffusa* or *T. aphrodisiaca*) has been documented a long history of use as a sexual tonic for several centuries in traditional Central and South American medicine, particularly within traditional Mexican herbal medicine, where it has been employed for centuries as an aphrodisiac, as well as for its stimulant and tonic effects on the nervous system. The aerial parts of the plant, mainly leaves and stems, are commonly used and are typically consumed as infusions or incorporated into herbal formulations [22,23,24] and are rich in flavonoids, particularly several C- and O-glycosides flavones and some methoxyflavones [25,26]. Preclinical studies in male rodents have reported pro-sexual effects of damiana extracts, including increased copulatory activity and reduced ejaculation latency [27,28,29,30]. Clinical evidence remains limited; however, small-scale studies and trials in combination formulations have reported improvements in sexual desire and satisfaction parameters [31,32,33]. Although the underlying molecular mechanisms are not fully elucidated, available data suggest the involvement of nitric oxide-related signaling pathways [29,30], phosphodiesterase-5 modulation, and aromatase regulation [34].

Despite these promising observations, the molecular mechanisms underlying the physiological effects of damiana remain insufficiently understood, and robust evidence linking phytochemical composition to biological activity is still lacking. Consequently, comprehensive studies integrating chemical characterization with mechanistic in vitro evidence are needed to properly assess both the quality and efficacy of these products. Addressing this gap, the present study provides an integrative evaluation of a commercial *T. diffusa* (Liboost^®^) extract specifically formulated to support sexual health, combining detailed phytochemical characterization with the exploration of potential molecular mechanisms.

## 2. Results and Discussion

### 2.1. Phytochemical Characterization of Turnera diffusa Extract

A detailed phytochemical characterization was conducted to define the chemical profile of the commercial *T. diffusa* extract. Particular emphasis was placed on the identification of flavonoids, given their reported association with biological activities relevant to sexual health [35,36,37,38].

The chemical profile of the commercial *T. diffusa* extract (Liboost^®^) was established using HPLC-DAD-HRMS, enabling both the identification and quantification of its major constituents. A total of 49 compounds were detected, predominantly belonging to the flavonoid class. The DAD chromatogram at 340 nm is presented in Figure S1, where compounds are numbered according to their corresponding entries in Table 1.

Flavones and flavonols constituted the predominant subclasses in the extract, with numerous luteolin- and apigenin-derived glycosides together with several methoxyflavones detected. The fragmentation patterns of flavonoids C- and O-glycosides present in *T. diffusa* have been previously investigated by Bernardo et al. [39]. In most cases, the fragment ions observed in the present study are consistent with those previously reported, thereby supporting and confirming the previously proposed compound identifications. Since the fragmentation pathways of these compounds have already been discussed in detail by these authors, only newly observed fragment ions, as well as compounds not previously identified in this matrix or whose fragmentation has not been previously addressed, are discussed below.

Among flavone glycosides, C-glycosyl flavones were particularly abundant, consistent with previous reports on *T. diffusa*. Their identification was supported by characteristic fragmentation features, including the typical cross-ring cleavages of C-linked sugars, leading to neutral losses of 90 and 120 Da for C-hexosides and 104 Da for C-deoxyhexosides. In addition, diagnostic fragment ions corresponding to the aglycone retaining part of the sugar moiety were observed, including aglycone +113 and aglycone +83 for C-diglycosides, and aglycone +71 and aglycone +41 for C-monoglycosides. Furthermore, in those compounds bearing additional O-glycosidic linkages, neutral losses associated with these substituents were also detected, namely 180 Da for O-hexoses, 164 Da for O-deoxyhexoses, and 118 Da for ketohexoses. These fragmentation patterns were consistently observed across the detected compounds and provided key support for their structural annotation.

Only two di-C-glycosides were observed, namely apigenin-6,8-di-C-glucoside (**1**) and acacetin-6,8-di-C-glucoside (**6**), whereas mono-C-glycosides were more prevalent, especially those derived from luteolin. Luteolin-8-C-(2-rhamnosyl) ketodihexoside (**24**), the most frequently reported luteolin-C-glycoside in *T. diffusa*, was confirmed in the present sample. Additional luteolin-C-glycosides included luteolin-8-C-(2-rhamnosyl)glucoside (**3**), luteolin-8-C-(2-rhamnosyl)quinivoside (**21**), a luteolin-C-(deoxyhexoside) hexoside isomer (**31**), and two luteolin-C-(deoxyhexoside) deoxyhexoside isomers (**30**, **32**), all of which have been previously described in damiana extracts [29,37,38,39,40].

In contrast, apigenin-C-glycosides were less diverse, with only apigenin-8-C-glucoside (**4**) and apigenin-8-C-(2-rhamnosyl) glucoside (**5**) detected. Notably, apigenin-8-C-glucoside (vitexin) has not been previously reported in *T. diffusa* as a single extract but only in combination with other botanical species [41]. In this case, the observation of the characteristic aglycone +71, aglycone +41 and aglycone +41 − H_2_O fragment ions, consistent with the typical −90/−120 Da cleavages described above, together with the use of an analytical standard, further supported its identification.

A different trend was observed for O-glycosyl flavones, where apigenin derivatives were more structurally diverse than luteolin derivatives. Their identification was supported by the characteristic cleavage of O-glycosidic bonds, leading to the release of the corresponding aglycones after the loss of sugar moieties, mainly as neutral losses of 162 Da (hexose). In the case of rhamnose-containing diglycosides, an additional neutral loss of 164 Da, corresponding to rhamnose with water elimination, was also observed. These fragmentation patterns enabled the confident annotation of several compounds within this subclass. Only luteolin-7-O-glucoside (**10**) was detected, representing, to the best of our knowledge, the first report of this compound in a damiana extract. Apigenin-7-O-glucoside (**14**) and two apigenin-7-O-(2-rhamnosyl) ketodeoxyhexoside isomers (**27**, **29**) were identified, in agreement with previous findings [37,39]. In addition, fragment ions at *m*/*z* 299 and 283 were detected for compound **17**, whereas compounds **22** and **25** showed fragment ions at *m*/*z* 473, 329 and 313. These ions were consistent with sequential losses involving water (*m*/*z* 473), the loss of the glucose moiety leading to the aglycone (*m*/*z* 329 for tricin and *m*/*z* 299 for chrysoeriol), and the subsequent loss of a methyl group from the aglycone (*m*/*z* 313 and 283, respectively). These fragmentation features supported the assignment of compound **17** as chrysoeriol-7-O-glucoside and compounds **22** and **25** as tricin-7-O-glucoside isomers, which have been previously detected in *T. diffusa* by Zhao et al. [37]. Five apigenin-7-O-coumaroylglucoside isomers (**39**, **41**, **42**, **44**, **45**) were also observed, representing the most characteristic apigenin derivatives reported for *T. diffusa* [7,29,30,31,32,33,34,35,36,37,38,39,40]. In these compounds, the fragment ion at *m*/*z* 431, corresponding to the loss of the coumaroyl moiety, was consistently detected, as previously reported [39]. Additionally, all isomers showed a fragment ion at *m*/*z* 145, corresponding to the p-coumaroyl anion, further supporting their structural assignment. Bernardo et al. [39] previously described four positional isomers corresponding to C4 and C6 coumaroyl linkages to the glucoside moiety, including both E and Z configurations. In the present extract, a fifth isomer was detected and tentatively assigned to a C3-linked coumaroyl glucoside, a structure previously reported in other plant species [39,42].

Flavonols were present in the sample exclusively as O-glycosides. Some of the detected flavonols had been confirmed in this plant species by previous studies; namely, myrcetin-3-O-(6-glucosyl)glucoside (**2**), Quercetin-3-O-(6-glucosyl)glucoside (**7**), larcitrin-3-O-(6-glucosyl)glucoside (**8**) and syringetin-3-O-(6-glucosyl)glucoside (**16**) [37,38,40]. Others flavonols like quercetin-3-O-(6-rhamnosyl) glucose (**9**), quercetin-3-O-glucoside (**11**), isorhamnetin-3-O-(6-glucosyl) glucoside (**13**) and quercetin-3-O-(2-rhamnosyl) ketodeoxyhexoside (**20**) had been previously reported only by Bernardo et al. [39] and were confirmed by this study. For these previously described compounds, the expected fragment ions corresponding to the aglycones were observed after neutral loss of the glycosidic moieties, in agreement with the fragmentation patterns. Finally, other flavonols were detected hitherto in this sample, tentatively identified as kaempferol-3-O-(2″-rhhamnosyl-galactoside)7-O-rhamnoside (**12**), quercetin-7-O-glucoside (**15**), isorhamnetin-3-O-glucoside (**18**), and diosmetin-8-C-(rhamnosyl)-glucoside (**19**). Their identification was supported by a fragmentation pattern consistent with that described for previously reported flavonols, characterized by the detection of aglycone ions following the neutral loss of glycosidic moieties. In particular, diagnostic fragment ions at *m*/*z* 301, 315 and 299 were observed, corresponding to quercetin, isorhamnetin and diosmetin aglycones, respectively. Additionally, diosmetin-8-C-(rhamnosyl)-glucoside showed a fragment ion at *m*/*z* 476, consistent with the neutral loss of the terminal rhamnose moiety, further supporting its structural assignment.

The corresponding flavone aglycones were also detected in their free, methoxylated, and prenylated forms. Free flavones such as luteolin (**28**) and apigenin (**35**) were observed, consistent with earlier reports [29,39]. Kaempferol (**33**) was the only flavonol aglycone detected and, to the best of our knowledge, is described here for the first time in *T. diffusa*, although kaempferol glycosides have been reported in the related species *T. subulata* [43]. Flavanones were also present, namely naringenin (**26**) and pinocembrin (**38**), previously reported in damiana by Willer et al. [38] and Zhao et al. [37], respectively. These assignments were supported by characteristic fragmentation patterns. In particular, fragment ions derived from Retro-Diels–Alder (RDA) cleavage of the C-ring were observed, including *m*/*z* 151 associated with A-ring cleavage, together with diagnostic B-ring fragments (^1^,^3^B^−^), which enabled the identification of luteolin (*m*/*z* 133), apigenin (*m*/*z* 117), and naringenin (*m*/*z* 119), in agreement with fragmentation pathways widely described in the literature [44,45]. In contrast, pinocembrin showed a distinct fragmentation behavior, characterized by the absence of CO losses and the presence of fragment ions at *m*/*z* 213 and 211, corresponding to neutral losses of C_2_H_2_ and CO_2_, respectively, which are typical of flavanones [44,46].

Regarding methoxylated derivatives, characteristic methoxyflavones of damiana such as acacetin (**47**), genkwanin (**48**), and velutin (**49**) were identified [29,37,38,39]. Acacetin and genkwanin, which are positional isomers, showed highly similar fragmentation patterns, sharing the main fragment ions. In both cases, a characteristic neutral loss of a methyl group was observed, yielding fragment ions at *m*/*z* 268, together with a common loss of CO (*m*/*z* 240) and a subsequent loss of CO (*m*/*z* 211). In contrast, velutin exhibited analogous fragmentation pathways, including the loss of one (*m*/*z* 298) and two methyl groups (*m*/*z* 283), as well as CO loss (*m*/*z* 255). Despite their similar fragmentation behavior, acacetin and genkwanin could be distinguished by the presence of a diagnostic fragment at *m*/*z* 165 in genkwanin, corresponding to a methylated A-ring, whereas acacetin only showed the fragment at *m*/*z* 151, corresponding to the non-methylated A-ring. Additionally, compound **37** with [M − H]^−^ *m*/*z* 299.0562 showed fragment ions corresponding to the neutral loss of a methyl group, followed by two consecutive CO losses (*m*/*z* 256 and 227), together with characteristic luteolin-derived fragments at *m*/*z* 151 and 133. Based on this fragmentation pattern, it was tentatively assigned to luteolin-7-methyl ether (3′,4′,5-trihydroxy-7-methoxyflavone), which is here reported for the first time in *T. diffusa*.

Prenylated (alkylated) flavone derivatives, including luteolin-8-C-E-propenoic acid (**40**), apigenin-8-C-propiolic acid (**43**), and a dihydroxyflavone-8-C-propiolic acid (**46**), had been previously reported in *T. diffusa* [39]; however, their fragmentation patterns have not been discussed to date. In all cases, fragment ions corresponding to the neutral loss of CO_2_ were observed, yielding *m*/*z* 311 for compound (**40**), *m*/*z* 293 for compound (**43**), and *m*/*z* 277 for compound (**46**). Additionally, luteolin-8-C-E-propenoic acid (**40**) showed a fragment ion at *m*/*z* 337, consistent with the loss of water, and the dihydroxyflavone-8-C-propiolic acid showed a fragment ion at *m*/*z* 293, consistent with a CO loss. For apigenin-8-C-propiolic acid (**43**), additional fragment ions at *m*/*z* 203 and 159 were detected, corresponding to the A-ring fragment bearing its hydroxyl groups plus the propiolic acid moiety, and its subsequent CO loss, respectively. The same fragment ions were also observed for compound (**46**), indicating a similar substitution pattern in the A-ring. This suggests that compound (**46**) contains two hydroxyl groups in the A-ring, as in apigenin. Considering that a dihydroxyflavone with two hydroxyl groups in the A-ring corresponds to chrysin (5,7-dihydroxyflavone), compound (**46**) was tentatively assigned as chrysin-8-C-propiolic acid (**46**).

Two additional luteolin derivatives (**34,36**) with [M − H]^−^ *m*/*z* 411.0794 were detected, consistent with compounds previously reported by Bernardo et al. [39] based on their quasi-molecular ions, although their fragmentation patterns were not described. These compounds showed fragment ions at *m*/*z* 383, corresponding to the loss of CO, followed by a fragment at *m*/*z* 337 resulting from the consecutive loss of an additional CO and H_2_O, as well as a fragment at *m*/*z* 285 corresponding to the luteolin aglycone after the loss of the unknown substituent. This fragmentation pattern further confirmed the proposed aglycone structure; however, it did not allow the identification of the side chain.

Regarding the quantitative results (Table 2), the total flavonoid content in the standardized commercial extract analyzed in this study was 15.9 mg·g^−1^, which is comparable to the values reported by Bernardo et al. [39] for an aqueous extract of *T. diffusa* (18.2 mg·g^−1^). Although the concentrations of most individual compounds were within similar ranges, notable differences were observed for certain subclasses, likely reflecting the use of solvents with different polarities as well as the intrinsic variability of the raw plant material.

The most abundant compound in the analyzed sample was luteolin-8-C-(2-rhamnosyl) ketodihexoside (3.00 mg·g^−1^), in agreement with previous reports [39,47]. Together with the remaining luteolin-C-glycosides, this subclass accounted for approximately 26% of the total flavonoid content (4.07 mg·g^−1^). The second most abundant compound was apigenin-8-C-propiolic acid (1.97 mg·g^−1^). When combined with luteolin-8-C-E-propenoic acid (0.52 mg·g^−1^) and dihydroxyflavone-8-C-propiolic acid (0.39 mg·g^−1^), C-prenylated flavones represented approximately 18% of the total flavonoids.

Apigenin-coumaroylglucoside isomers (1.72 mg·g^−1^) and flavonol glycosides (1.59 mg·g^−1^) each contributed close to 10% of the total flavonoid fraction. Methoxyflavones including acacetin, genkwanin, velutin, and luteolin-7-methyl ether accounted for 0.52 mg·g^−1^, while free flavones such as apigenin (0.29 mg·g^−1^), luteolin (0.14 mg·g^−1^), and pinocembrin (0.08 mg·g^−1^) were present at lower concentrations.

In contrast, in the aqueous extract analyzed by Bernardo et al. [39], luteolin-C-glycosides (11.58 mg·g^−1^) represented more than 60% of the total flavonoids, whereas apigenin-coumaroylglucoside derivatives (0.58 mg·g^−1^), prenylated flavones (0.59 mg·g^−1^), and methoxyflavones (0.04 mg·g^−1^) were minor constituents. Although the overall flavonoid content of both extracts was comparable, the commercial extract evaluated in the present study appears to be relatively enriched in less polar compounds, including methoxyflavones, prenylated flavones, and apigenin-coumaroylglucoside isomers.

These differences underscore the influence of the extraction process on the phytochemical profile of *T. diffusa* preparations, which may ultimately impact their bioactive properties and physiological effects.

### 2.2. In Vitro Evaluation of Biological Activity

To complement the chemical characterization, the biological activity of the extract was assessed through a series of mechanistic in vitro assays targeting pathways implicated in sexual function. This integrative approach aimed to provide functional context for the detected phytochemicals.

Importantly, cell viability assays performed in HepG2, NHDF, and HUVECs confirmed the absence of cytotoxic effects at the concentrations used in the mechanistic assays (Supplementary Tables S1 and S2), indicating that the observed biological responses were not attributable to nonspecific cellular damage. However, a reduction in HUVEC viability was observed at higher concentrations (400 and 1000 µg/mL), indicating growth inhibition outside the range selected for the NO assay. Therefore, the biological responses reported here are not attributable to nonspecific cellular damage under the experimental conditions employed (Supplementary Table S2).

The interpretation of the observed biological effects should consider the intrinsic complexity of botanical extracts. Unlike single-compound pharmacological studies, plant extracts such as *T. diffusa* contain multiple bioactive constituents that can act simultaneously on different molecular targets, often resulting in multi-target and synergistic effects [48]. In this context, the selection of a single pharmacological positive control may not adequately reflect the spectrum of mechanisms involved.

**Figure 1:**
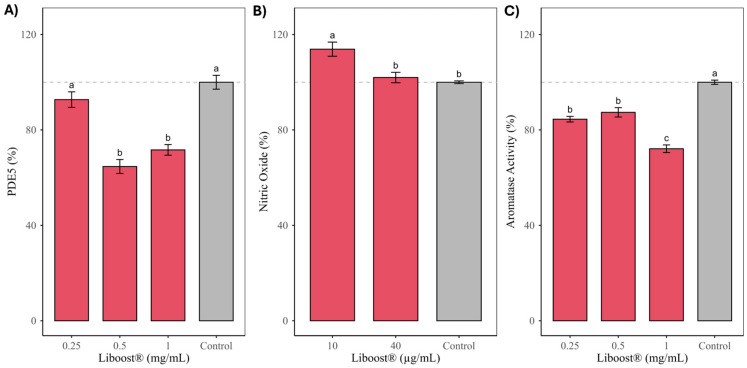
Effects of Liboost^®^ on (**A**) PDE5 expression, (**B**) nitric oxide (NO) production, and (**C**) aromatase enzymatic activity in cell-based assays. PDE5 expression levels following treatment with Liboost^®^ at 0.25, 0.5, and 1.0 mg/mL, expressed as percentage of the untreated control. Aromatase activity after exposure to Liboost^®^ at the same concentrations, expressed as percentage of control. NO production following treatment with Liboost^®^ at 10 and 40 µg/mL, expressed as percentage of control. Data are presented as mean ± SD. Different letters above bars indicate statistically significant differences among groups according to one-way ANOVA followed by post hoc analysis (*p* < 0.05). The dashed line represents control values (100%). Untreated cells were used as negative controls; LPS (0.2 µg/mL) was used as a positive control in the NO assay. Liboost^®^ significantly increased nitric oxide (NO) production (**B**), reaching 115% of control at 10 µg/mL, which was significantly higher than the untreated control. In contrast, NO levels at 40 µg/mL (101.87%) did not differ significantly. The involvement of NO signaling in the pro-sexual effects of *T. diffusa* has been robustly demonstrated in animal models, where the pro-sexual effects were abolished by nitric oxide synthase inhibition, confirming a key role for this pathway [29,30]. Comparable NO-dependent pro-sexual effects have also been reported for Chrysactinia mexicana, supporting a conserved mechanism among flavonoid-rich botanicals traditionally used as aphrodisiacs [54]. In addition, several flavonoids identified in Liboost^®^ (apigenin, luteolin, quercetin, pinocembrin, and myricetin) have been reported to enhance NO bioavailability predominantly through eNOS-mediated mechanisms, including increased enzyme activity and protection of NO from oxidative degradation [53,57,58,59,60]. In contrast, evidence for modulation of nNOS is more limited and appears to be largely restricted to luteolin, supporting a potential contribution to centrally mediated NO effects. Overall, the moderate increase in NO production observed here is consistent with a physiological, multi-level modulation of nitrergic signaling, integrating primarily peripheral vascular mechanisms with a more selective central component, in line with the reported NO-dependent pro-sexual effects of *T. diffusa* and related species.

Liboost^®^ significantly reduced PDE5 expression (Figure 1A), reaching approximately 65 and 75% of control levels at concentrations of 0.5 and 1.0 mg/mL, respectively, with these reductions being statistically significant compared with the control. Although PDE5 catalytic activity was not directly assessed, the observed downregulation of PDE5 expression is functionally relevant, as enzyme abundance is a major determinant of cellular cGMP turnover and NO–cGMP signaling efficiency in smooth muscle tissue [49]. A reduction in PDE5 levels is therefore expected to favor vasorelaxation and erectile signaling. This observation is consistent with extensive evidence indicating that flavonoids structurally related to those identified in Liboost^®^, particularly luteolin, apigenin, and quercetin, interact with PDE5 and modulate its function. Enzyme-based studies evaluating phosphodiesterase panels that include PDE5 have shown that luteolin and quercetin inhibit PDE5 at low micromolar concentrations, with reported IC_50_ values of approximately 3–6 µM for luteolin and 7–12 µM for quercetin, whereas apigenin displays weaker inhibition of PDE5, with IC_50_ values generally in the higher micromolar range (>10 µM), indicating a lower potency but still measurable interaction [50]. Although these studies primarily describe catalytic inhibition rather than regulation of enzyme expression, they establish a direct molecular interaction between these flavonoids and PDE5 that supports broader regulatory effects on this pathway. In silico analyses further corroborate these findings by demonstrating favorable binding of luteolin, quercetin, and apigenin within the catalytic pocket of PDE5, with predicted affinities consistent with moderate PDE5 modulation relative to synthetic inhibitors [51]. Importantly, functional studies using flavonoid-rich plant extracts have reported reductions in PDE5 levels and/or PDE5-related signaling accompanied by increased nitric oxide availability in biological systems, supporting the physiological relevance of flavonoid-mediated regulation of the PDE5–NO axis beyond direct enzymatic inhibition [52,53]. In vivo studies on *T. diffusa* and botanically related species further demonstrate that enhancement of sexual function is critically dependent on NO–cGMP signaling, as blockade of nitric oxide synthase abolishes pro-sexual effects, indicating that modulation of key components of this pathway, including PDE5 expression, is sufficient to produce functional outcomes [27,29,30,54]. From a translational perspective, comprehensive reviews emphasize that plant-derived flavonoids typically act as low-potency, multi-target modulators, influencing enzyme expression and signaling networks rather than functioning as high-affinity single-target inhibitors, with biological relevance emerging from additive and synergistic effects within complex botanical matrices [15,55]. Collectively, these data support the interpretation that the decrease in PDE5 expression observed with Liboost^®^ reflects a biologically meaningful modulation of the NO–cGMP pathway, consistent with the known pharmacology of its flavonoid constituents.

Also, this damiana extract significantly decreased aromatase enzymatic activity (Figure 1C), producing inhibitions of 15.49% and 12.63% at 0.25 and 0.5 mg/mL, respectively; both concentrations differ significantly from the control but also from each other. At 1.0 mg/mL, aromatase activity was reduced by 27.89%, representing a level of inhibition that was significantly greater than both the control and the lower concentrations. Aromatase plays a pivotal role in the conversion of androgens to estrogens, and increased aromatase activity has been associated with reduced androgen bioavailability and impaired sexual function [56]. The magnitude and concentration range of the inhibition observed here are consistent with phytochemical studies on *T. diffusa*, in which pinocembrin and acacetin were identified as the most potent aromatase inhibitors among isolated constituents, exhibiting IC_50_ values in the low micromolar range (approximately 10–11 µM for pinocembrin and 15–20 µM for acacetin) in enzyme-based assays [34,35]. In the same study, apigenin derivatives, including apigenin-7-O-glucoside, showed moderate but significant aromatase inhibition, indicating that multiple flavonoids present in damiana contribute additively to the overall effect. Beyond these compounds, other flavonoids structurally related to those identified in Liboost^®^, such as luteolin, quercetin, and myricetin, have been reported to exert weak to moderate modulatory effects on aromatase activity and broader estrogen-related pathways, generally at micromolar concentrations, although with lower potency and selectivity compared with pinocembrin and acacetin [53,57,58,59,60]. Overall, the moderate inhibition observed (13–28%) suggests a modest modulatory effect on aromatase activity, which is consistent with the relatively weak inhibitory potency generally reported for flavonoid-rich botanical extracts [15,55].

Collectively, the simultaneous modulation of PDE5 expression, aromatase activity, and NO production supports a convergent and potentially synergistic mechanism of action for Liboost^®^, integrating vascular, endocrine, and nitrergic components of sexual function. Rather than acting through a single high-affinity molecular target, Liboost^®^ appears to function as a multi-target physiological modulator, in which moderate effects on complementary pathways combine to produce biologically meaningful outcomes. This mode of action is consistent with current conceptual frameworks describing flavonoid-rich botanical supplements for sexual health, which emphasize coordinated, low-intensity modulation of interconnected signaling systems rather than pharmacological inhibition of a single pathway [15,27,55].

These interpretations are based on in vitro and cell-based assays, which, while mechanistically informative, do not fully reproduce the neuroendocrine and behavioral complexity of sexual function in vivo. In addition, although the flavonoid profile of Liboost^®^ provides a plausible explanation for the observed effects, the individual contribution of each compound and potential synergistic interactions were not specifically addressed. This is particularly relevant in the context of botanical extracts, which are known to exert their biological activity through multi-component and multi-target mechanisms rather than single-compound effects.

Furthermore, the concentrations tested in vitro may not directly reflect systemic exposure following oral intake, as bioavailability and metabolism were not evaluated. Therefore, in vivo studies and controlled clinical trials will be required to confirm the physiological relevance and translational potential of these findings.

A limitation of the present study is the absence of assay-specific pharmacological positive controls in all experimental systems, which may limit direct comparison with reference compounds. However, the study was designed as an initial functional screening to identify potential biological activities of the extract. In this context, the selection of a single positive control is inherently challenging due to the complexity of both the biological processes involved and the multi-component nature of the extract.

It should also be considered that, due to the complex composition of the extract, the effective concentration of individual bioactive compounds is substantially lower than the total extract concentration. In the present study, the extract is standardized to contain approximately 1.5% total flavonoids. Therefore, the highest concentration tested in the NO production assay (40 µg/mL extract) corresponds to approximately 0.6 µg/mL of flavonoids, while the maximum concentration used in the PDE5 and aromatase inhibition assays (1 mg/mL extract) corresponds to approximately 15 µg/mL of flavonoids. These values fall within biologically relevant ranges for flavonoids in in vitro studies, supporting the physiological relevance of the observed effects. Nevertheless, the possibility of nonspecific interactions at higher concentrations cannot be excluded and warrants further investigation using refined dose–response approaches.

## 3. Materials and Methods

### 3.1. Reagents and Standards

All solvents used for chromatographic analysis were of LC–MS grade. Water, methanol, and formic acid were purchased from Scharlab, (Barcelona, Spain). Reference standards of flavonoids and related phenolic compounds, including rutin, vitexin, luteolin-7-O-glucoside, quercetin-3-O-glucoside, apigenin-7-O-glucoside, isorhamnetin-3-O-glucoside, kaempferol, luteolin, and acacetin, were obtained from Sigma-Aldrich (St. Louis, MO, USA); while vitexin-2″-O-rhamnosyl and (+)-pinocembrin were acquired from Indagoo (Barcelona, Spain); vicenin II from Biopurify Phytochemicals (Sichuan, China) and apigenin from TCI (Tokyo, Japan). For the in vitro assays, the following kits were employed: a PDE5 activity assay kit from MyBioSource (San Diego, CA, USA), a nitric oxide detection kit Calbiochem^®^ from Merck Millipore (San Diego, CA, USA), and an aromatase activity assay kit from Abcam (Cambridge, UK). All assays were performed according to the manufacturer’s instructions. Cell culture reagents, including Dulbecco’s Modified Eagle Medium (DMEM), fetal bovine serum (FBS), penicillin–streptomycin solution, and trypsin–EDTA, were obtained from Thermo Fisher Scientific, Waltham, MA, USA.

### 3.2. Cell Cultures

Three human cell lines were used for the in vitro experiments: HUVEC (85011430) supplied by the European Collection of Authenticated Cell Cultures (ECACC; Salisbury, UK), HepG2 obtained from the Centro de Instrumentación Científica (CIC) of the University of Granada (Granada, Spain), and NHDF (Cat. No. C-12302) provided by PromoCell GmbH (Heidelberg, Germany). All cell types were maintained at standard conditions (37 °C, 5% CO_2_). Human umbilical vein endothelial cells (HUVECs), used for nitric oxide production and WST-1 cytotoxicity assays, were cultured in F12K medium supplemented with 1.5 g/L sodium bicarbonate, 0.10 mg/mL heparin, 0.03 mg/mL endothelial cell growth supplement, and 10% fetal bovine serum (FBS). HepG2 hepatocytes, used for aromatase inhibition studies, were grown in Eagle’s Minimum Essential Medium (EMEM) containing 10% FBS and 1% penicillin–streptomycin. Neonatal human dermal fibroblasts (NHDFs), used for phosphodiesterase-5 (PDE-5) inhibition assays, were cultured in Dulbecco’s Modified Eagle Medium (DMEM) supplemented with 10% FBS and 1% penicillin–streptomycin.

### 3.3. Samples and Sample Preparation

The sample investigated in this study consisted of Liboost^®^, a commercial hydroalcoholic extract of damiana (*T. diffusa*), manufactured by Pharmactive Biotech Products S.L.U. (Madrid, Spain). The extract is standardized to contain more than 1.5% of total flavonoids, under the commercial name of Liboonoides^®^, as declared by the manufacturer. The sample was stored under dry conditions at room temperature until analysis.

Prior to analysis, the extract was homogenized using a spatula to ensure uniformity. Approximately 80 mg of sample were accurately weighed into a 5 mL volumetric flask and dissolved in dimethyl sulfoxide (DMSO). The flask was filled to the calibration mark with DMSO (final volume 5 mL). The solution was vortex-mixed for 30 s, followed by sonication for 10 min to ensure complete dissolution. The resulting solution was filtered through a 0.22 μm membrane filter before chromatographic analysis.

### 3.4. HPLC-DAD-HRMS Analysis

The system employed for the analysis of the sample was an Agilent 1260 Infinity II Prime LC System with a diode array detector (DAD) coupled to a 6230B time-of-flight (ToF) MS detector provided with an Agilent Jet Stream Electrospray Ionization (AJS-ESI) source (Agilent Technologies, Santa Clara, CA, USA). Analysis was carried out according to the method developed by Benito-Vazquez et al. [61]. In summary, chromatographic analysis was performed on a Kinetex F5 column (100 × 2.1 mm, 2.6 µm, 100 Å; Phenomenex, Torrance, CA, USA) at 30 °C using water (eluent A) and methanol (eluent B), both with 0.1% formic acid, and following this gradient: 10% B (0 min), 33% B (3 min), 33% B (7 min), 37% B (16 min), 49% B (22 min), 56% B (37 min), 94% B (43 min) and 10% B (48 min) at a 0.5 mL/min flow.

Compound identification was based on UV spectra, chromatographic retention, and exact mass spectrometry data, which was confirmed, when possible, by co-injection of the corresponding commercial standards (Section 3.1). In addition, a run was completed with high fragmentor voltage conditions (up to 250 V) applied to promote in-source fragmentation, allowing the acquisition of additional diagnostic fragment ions complementary to the rest of the data. The identifications were considered tentative when analytical standards were not available. For quantitative analysis, absorbance was measured at 340 nm and an external standard calibration curve of rutin (0.2–50 ppm) was used for the quantification of diglycosidic flavonoids, an external standard calibration curve of luteolin-7-O-glucoside (0.4–25 ppm) was used for the quantification of monoglycosidic and monoprenylated flavonoids and an external standard calibration curve of acacetin (0.2–20 ppm) was used for the quantification of aglycones and methylated flavonoids. Data acquisition and processing were performed using MassHunter Software (v.10.1.48, Agilent Technologies, Santa Clara, CA, USA).

### 3.5. Cell Proliferation/Citotoxicity Assays

Cytotoxicity of Liboost^®^ was evaluated in all cell lines prior to their corresponding functional assays. For the NO synthesis assay, HUVECs were cultured at 37 °C in a humidified 5% CO_2_ atmosphere and then exposed to a concentration range of Liboost^®^ from 1000 to 10 µg/mL. After treatment, cell morphology was examined by bright-field microscopy, and WST-1 reagent was added to each well and incubated for 2 h. Absorbance was subsequently measured at 400 nm with a 600 nm reference wavelength, and cell viability was calculated relative to untreated controls (defined as 100%). For the aromatase and PDE-5 inhibition assays, cytotoxicity was assessed in HepG2 hepatocytes and NHDFs, respectively, which were cultured and maintained under the same standard conditions. These cells were exposed to a concentration range of Liboost^®^ from 0.008 to 1.0 mg/mL, followed by incubation with MTT (1 mg/mL) for 3 h. After incubation, the MTT solution was removed, and the resulting formazan crystals were dissolved in 100 µL of isopropanol. Absorbance was measured at 540 nm, and cell viability was expressed relative to untreated controls.

### 3.6. Nitric Oxide Production Assay

Cells were exposed to Liboost^®^ at 40 and 10 µg/mL (non-cytotoxic concentrations), and to lipopolysaccharide (LPS, 0.2 µg/mL) as a positive control, for 24 h. Following incubation, culture supernatants were collected and nitric oxide levels were quantified using a Nitric Oxide Assay Kit (Cat. No. 482650, Merck, San Diego, CA, USA), based on enzymatic reduction in nitrate to nitrite followed by detection with the Griess reagent [62]. Absorbance was measured at 540 nm according to the manufacturer’s instructions, and NO concentrations (µM) were calculated by interpolation from a standard curve generated with sodium nitrite standards included in the kit.

### 3.7. Aromatase Inhibiton Assay

Non-cytotoxic concentrations of Liboost^®^ (1.0, 0.5, and 0.25 mg/mL) were selected for HepG2 incubation. Aromatase activity was quantified using the Abcam fluorometric aromatase kit (ab273306), which converts a fluorogenic substrate into a highly fluorescent metabolite detected at Ex/Em = 488/527 nm. Following the manufacturer’s instructions, a fluorescence standard curve was generated from serial dilutions of the provided Aromatase Fluorescence Standard, enabling conversion of relative fluorescence units into pmoles of metabolized substrate. After incubation with Liboost^®^, fluorescence was measured using a microplate reader, and aromatase activity was calculated by interpolating sample values against the standard curve. Final activity values were expressed relative to untreated control cells.

### 3.8. Phosphodiesterease 5 (PDE-5) Inhibition Assay

Cells were treated with Liboost^®^ at 1.0, 0.5, and 0.2 mg/mL, and PDE-5 levels in the culture supernatants were quantified using a Human Phosphodiesterase 5 (PDE5) ELISA Kit (Cat. No. MBS3801839, MyBioSource, San Diego, CA, USA). After treatment, the conditioned medium was transferred to antigen-coated wells, incubated with the HRP-conjugate reagent, and washed according to the manufacturer’s instructions. Chromogenic substrates A and B were then added, and the reaction was developed at 37 °C in the dark before being stopped to allow color stabilization. The intensity of the resulting signal was proportional to the amount of PDE-5 present and was measured spectrophotometrically at 450 nm. PDE-5 concentrations were calculated by interpolating sample absorbance values from a standard curve provided with the kit, and results were normalized to untreated controls.

### 3.9. Controls and Experimental Design

Untreated cells were used as negative controls in all assays to establish basal response levels. For the nitric oxide (NO) production assay, lipopolysaccharide (LPS, 0.2 µg/mL) was included as a positive control to confirm assay responsiveness. No additional pharmacological positive controls were included in the remaining assays, as the experimental design was intended for exploratory screening of the biological activity of the extract rather than for quantitative benchmarking against reference compounds. The tested Liboost^®^ concentration range was selected based on preliminary screening studies aimed at identifying biologically active, non-cytotoxic conditions. Cell viability assays confirmed that all tested concentrations were non-cytotoxic under the experimental conditions.

### 3.10. Statistical Analysis

Data are expressed as mean ± standard error of the mean (SEM) from independent experiments. Statistical analyses were performed using R software (version 4.5.1). For each experimental model (PDE5 inhibition, aromatase inhibition, and nitric oxide activation), differences among treatment concentrations were analyzed using one-way analysis of variance (ANOVA). When the ANOVA model showed statistical significance, pairwise comparisons were conducted using Tukey’s Honestly Significant Difference (HSD) post hoc test. Statistical differences between groups are indicated by different letters. A *p*-value < 0.05 was considered statistically significant.

## 4. Conclusions

The present study provides an integrated chemical and mechanistic evaluation of a commercial *T. diffusa* extract (Liboost^®^) associated with sexual health support. Comprehensive HPLC-DAD-HRMS analysis revealed a complex flavonoid-rich profile dominated by luteolin and apigenin derivatives, methoxyflavones, and flavanones, which are compounds previously linked to vascular and endocrine modulation. Functional assays in human cell models demonstrated that the extract simultaneously reduced PDE5 expression, inhibited aromatase activity, and moderately increased nitric oxide production without inducing cytotoxicity. These complementary effects support a multi-target mode of action involving key pathways that regulate sexual function, particularly the NO-cGMP signaling axis and androgen–estrogen balance. Although the magnitude of the individual effects was moderate, their convergence suggests that flavonoid-rich botanical matrices such as *T. diffusa* may act as physiological modulators rather than single-target pharmacological inhibitors. Overall, these findings contribute mechanistic evidence linking the phytochemical composition of damiana extracts with biological activities relevant to sexual health, while highlighting the need for further in vivo and clinical studies to confirm their physiological relevance and therapeutic potential.

## Figures and Tables

**Table 1:** Identified flavonoids by HPLC-MS (TOF) in commercial damiana extract (Liboost^®^).

No.	Compound	Rt (min)	Molecular Formula	MW	Detected *m*/*z* Ions	Fragments (*m*/*z*)	Mass Error (ppm)	Standard
**1**	Apigenin-6,8-di-C-glycoside (Vincenin II)	5.137	C_27_H_30_O_15_	594.1585	593.1512 [M − H]^−^	473.1097	0.01	Yes
						353.0693		
	
**2**	Myrcetin-3-O-(6-glucosyl)glucoside	5.937	C_27_H_30_O_18_	642.1432	641.1357 [M − H]^−^	316.0232	−0.42	No
	
	
**3**	Luteolin-8-C-(rhamnosyl)glucoside	6.22	C_27_H_30_O_15_	594.1585	593.1511 [M − H]^−^	473.1096	−0.27	No
					693.1583 [M + HCOO]^−^	357.0626		
						327.0522		
**4**	Apigenin-8-C-glucoside (Vitexin)	6.729	C_21_H_20_O_10_	432.1056	431.0985 [M − H]^−^	341.0585	0.3	Yes
						311.0502		
						293.0472		
**5**	Apigenin-8-C-(rhamnosyl) glucoside (Vitexin-2″-O-rhamnosyl)	6.854	C_27_H_30_O_14_	578.1636	577.1561 [M − H]^−^	457.1151	−0.26	Yes
					623.1661 [M + HCOO]^−^	413.0890		
						293.0468		
**6**	Acacetin-6,8-di-C-glucoside	6.904	C_28_H_32_O_15_	608.1741	607.1667 [M − H]^−^	487.1256	−0.59	No
					653.1859 [M + HCOO]^−^			
	
**7**	Quercetin-3-O-(6-glucosyl)glucoside	7.062	C_27_H_30_O_17_	626.1483	625.1408 [M − H]^−^	301.1238	−0.53	No
					671.1271 [M + HCOO]^−^			
	
**8**	Larcitrin-3-O-(6-glucosyl)glucoside	8.229	C_27_H_30_O_16_	656.1589	655.1520 [M − H]^−^	331.0407	0.42	No
	
	
**9**	Quercetin-3-O-(6-rhamnosyl)-glucoside (Rutin)	8.787	C_27_H_30_O_16_	610.1534	609.1458 [M − H]^−^	301.0364	−0.51	Yes
	
	
**10**	Luteolin-7-O-glucoside	8.879	C_27_H_30_O_16_	448.1006	447.0931 [M − H]^−^	285.0350	−0.07	Yes
					493.0981 [M + HCOO]^−^			
	
**11**	Quercetin-3-O-glucoside	8.896	C_21_H_20_O_12_	464.0955	463.0879 [M − H]^−^	301.0313	−0.58	Yes
	
	
**12**	Kaempferol-3-O-(2″-rhhamnosyl-galactoside)7-O-rhamnoside	9.271	C_33_H_40_O_19_	740.2164	739.2084 [M − H]^−^		−1.71	No
					785.2087 [M + HCOO]^−^			
	
**13**	Isorhamnetin-3-O-(6-glucosyl)glucoside	10.221	C_28_H_32_O_17_	640.1639	639.1564 [M − H]^−^	315.0513	−0.8	No
					685.1607 [M + HCOO]^−^			
	
**14**	Apigenin-7-O-glucoside	11.471	C_21_H_20_O_10_	432.1056	431.0983 [M − H]^−^	269.0447	−0.19	Yes
					477.1056 [M + HCOO]^−^			
	
**15**	Quercetin-7-O-glucoside	11.662	C_21_H_20_O_12_	464.0955	463.0879 [M − H]^−^	301.0299	−0.79	No
					509.1012 [M + HCOO]^-^			
	
**16**	Syringetin-3-O-(6-glucosyl)glucoside	12.129	C_29_H_34_O_18_	670.1745	669.1667 [M − H]^−^	345.0566	−1.52	No
					715.1530 [M + HCOO]^−^			
	
**17**	Chrysoeriol-7-O-glucoside	12.829	C_22_H_22_O_11_	462.1162	461.1087 [M − H]^−^	299.0485	−0.58	No
					507.1131 [M + HCOO]^−^	283.0195		
	
**18**	Isorhamnetin-3-O-glucoside	12.966	C_22_H_22_O_12_	478.1111	477.1035 [M − H]^−^	315.0474	−0.25	Yes
					523.1098 [M + HCOO]^−^			
	
**19**	Diosmetin-8-C-(rhamnosyl)-glucoside	13.113	C_28_H_32_O_15_	608.1741	607.1667 [M − H]^−^	461.1059	−1.23	No
						299.0501		
	
**20**	Quercetin-3-O-(2-rhamnosyl)ketodeoxihexoside	13.771	C_27_H_28_O_15_	592.1428	591.1351 [M − H]^−^	427.0676	−0.52	No
					637.1289 [M + HCOO]^−^	301.0358		
	
**21**	Luteolin-8-C-(rhamnosyl)quinovoside	13.929	C_27_H_30_O_14_	578.1636	577.1556 [M − H]^−^	473.1097	−1.34	No
					623.1549 [M + HCOO]^−^	413.0876		
						357.0618		
						327.0516		
**22**	Tricin-7-O-glucoside	14.096	C_22_H_22_O_11_	492.1268	491.1191 [M − H]^−^	473.1068	−0.96	No
					537.1283 [M + HCOO]^−^	329.0511		
						313.0329		
**23**	Luteolin-8-C-(2-deoxihexoside)-ketodeoxihexoside	15.938	C_27_H_28_O_14_	576.1479	575.1407 [M − H]^−^	411.0729	0.12	No
						285.0414		
	
**24**	Luteolin-8-C-(2-rhamnosyl)-ketodeoxihexoside	17.738	C_27_H_28_O_14_	576.1479	575.1403 [M − H]^−^	411.0726	−0.57	No
						285.0412		
	
**25**	Tricin-glucoside	18.405	C_23_H_24_O_12_	492.1268	491.1192 [M − H]^−^	473.1094	−0.86	No
					537.1267 [M + HCOO]^−^	313.0368		
	
**26**	Naringenin	19.038	C_15_H_12_O_5_	272.0685	271.0610 [M − H]^−^	151.0047	−0.92	No
					317.0631 [M + HCOO]^−^	119.0511		
						107.0150		
**27**	Apigenin-7-O-(2-rhamnosyl)ketodeoxihexoside	19.938	C_27_H_28_O_13_	560.153	559.1453 [M − H]^−^	395.0788	−0.48	No
					605.1458 [M + HCOO]^−^	269.0468		
	
**28**	Luteolin	20.921	C_15_H_12_O_5_	286.0477	285.0404 [M − H]^−^	199.0337	−0.92	No
					331.0459 [M + HCOO]^−^	175.0336		
						150.9977		
						133.0238		
**29**	Apigenin-7-O-(2-rhamnosyl)ketodeoxihexoside	21.146	C_27_H_28_O_13_	560.153	559.1458 [M − H]^−^	395.0797	−0.1	No
					605.1444 [M + HCOO]^−^	269.0487		
	
**30**	Luteolin-C-(deoxihexoside)deoxihexoside	21.672	C_27_H_28_O_14_	576.1479	575.1403 [M − H]^−^	411.0738	−0.5	No
					621.1522 [M + HCOO]^−^	285.0417		
	
**31**	Luteolin-C-(deoxihexoside)hexoside	22.338	C_27_H_28_O_15_	592.1428	591.1354 [M − H]^−^	473.1085	−0.44	No
					637.1227 [M + HCOO]^−^	327.0498		
	
**32**	Luteolin-C-(deoxihexoside)deoxihexoside	22.560	C_27_H_28_O_14_	576.1479	575.1406 [M − H]^−^	411.0701	−0.27	No
					621.1318 [M + HCOO]^−^	285.0381		
	
**33**	Kaempferol	22.922	C_15_H_12_O_5_	286.0477	285.0403 [M − H]^−^		−0.89	Yes
					331.0442 [M + HCOO]^−^			
	
**34**	Luteolin derivate	23.630	C_21_H_16_O_9_	412.0794	411.0722 [M − H]^−^	383.0803	−0.79	No
						337.0370		
						297.0412		
						285.0447		
**35**	Apigenin	24.047	C_15_H_10_O_5_	270.0528	269.0454 [M − H]^−^	227.0363	−0.79	Yes
					315.0434 [M + HCOO]^−^	151.0460		
						117.0357		
**36**	Luteolin derivate	24.805	C_21_H_16_O_9_	412.0794	411.0726 [M − H]^−^	383.0794	1.08	No
						337.0377		
						297.0417		
						285.0416		
**37**	Luteolin methyl ether	25.230	C_16_H_12_O_6_	300.0634	299.0562 [M − H]^−^	285.0362	0.3	No
						256.0349		
						227.0328		
						151.0010		
						133.0269		
**38**	Pinocembrin	25.847	C_15_H_12_O_4_	256.0736	255.0662 [M − H]^−^	213.0565	−0.6	Yes
						211.0768		
						151.0045		
**39**	Apigenin-7-O-(4″-p-E-coumaroyl)glucoside	26.105	C_30_H_26_O_12_	578.1424	577.1352 [M − H]^−^	431.0986	0.17	No
					623.1401 [M + HCOO]^−^	269.0466		
						145.0304		
**40**	Luteolin-8-C-E-propenoic acid	26.464	C_18_H_12_O_8_	356.0532	355.0456 [M − H]^−^	337.0368	−0.74	No
						311.0567		
	
**41**	Apigenin-7-O-(6″-p-E-coumaroyl)glucoside	26.697	C_30_H_26_O_12_	578.1424	577.1349 [M − H]^−^	431.0995	−0.56	No
						269.0465		
						145.0304		
**42**	Apigenin-7-O-(6″-p-Z-coumaroyl)glucoside	27.214	C_30_H_26_O_12_	578.1424	577.1348 [M − H]^−^	431.0981	−0.54	No
						269.0468		
						145.0302		
**43**	Apigenin-8-C-propiolic acid	27.689	C_18_H_12_O_8_	338.0427	337.0351 [M − H]^−^	293.0462	−0.62	No
					383.0408 [M + HCOO]^−^	202.9994		
						159.0096		
**44**	Apigenin-7-O-(4″-p-Z-coumaroyl)glucoside	27.957	C_30_H_26_O_12_	578.1424	577.1351 [M − H]^−^	431.0991	−0.27	No
					623.1306 [M + HCOO]^−^	269.0460		
						145.0303		
**45**	Apigenin-7-O-(3″-p-coumaroyl)glucoside	29.765	C_30_H_26_O_12_	578.1424	577.1354 [M − H]^−^	431.0949	0.43	No
						269.0461		
						145.0313		
**46**	Chrysin-8-C-propiolic acid	30.706	C_18_H_10_O_6_	322.0477	321.0402 [M − H]^−^	293.0462	−0.84	No
						277.0513		
						202.9994		
						159.0099		
**47**	Acacetin (5,7-Dihydroxy-4′-methoxyflavone)	31.772	C_16_H_12_O_5_	284.0682	283.0609 [M − H]^−^	268.0385	−1.05	Yes
						240.0434		
						211.0410		
						151.0056		
**48**	Genkwanin (4′,5-dihydroxy-7-methoxyflavone)	32.656	C_16_H_12_O_5_	284.0682	283.0610 [M − H]^−^	268.0396	−0.8	No
					329.0645 [M + HCOO]^−^	240.0435		
						211.0411		
						165.0212		
**49**	Velutin (5,4′-Dihydroxy-7,3′-dimethoxyflavone)	34.347	C_17_H_14_O_6_	314.079	313.0716 [M − H]^−^	298.0464	−0.73	No
						283.0229		
						255.0278		

**Table 2:** Concentrations of flavonoids in commercial damiana extract (Liboost^®^) by HPLC-DAD.

Nr	Compound	Concentration (mg·g^−1^)	Quantification Standard
**1**	Apigenin-6,8-di-C-glycoside (Vincenin II)	0.145 (0.001) *	Rutin
**2**	Myrcetin-3-O-(6-glucosyl)glucoside	0.163 (0.002)	Rutin
**3**	Luteolin-8-C-(rhamnosyl)glucoside	0.589 (0.008)	Rutin
**4**	Apigenin-8-C-glucoside (Vitexin)	0.135 (0.001)	Luteolin-7-O-glucoside
**5 + 6**	Apigenin-8-C-(rhamnosyl)glucoside Acacetin-6,8-di-C-glucoside	0.205 (0.003)	Rutin
**7**	Quercetin-3-O-(6-glucosyl)glucoside	0.148 (0.003)	Rutin
**8**	Larcitrin-3-O-(6-glucosyl)glucoside	0.341 (0.007)	Rutin
**9**	Quercetin-3-O-(6-rhamnosyl)-glucoside (Rutin)	0.064 (0.003)	Rutin
**10**	Luteolin-7-O-glucoside	0.139 (0.001)	Luteolin-7-O-glucoside
**11 +** **12**	Quercetin-3-O-glucoside Kaempferol-3-O-(2″-rhhamnosyl-galactoside)7-O-rhamnoside	0.0760 (0.0004)	Luteolin-7-O-glucoside
**13**	Isorhamnetin-3-O-(6-glucosyl)glucoside	0.177 (0.004)	Rutin
**14**	Apigenin-7-O-glucoside	0.18 (0.01)	Luteolin-7-O-glucoside
**15**	Quercetin-7-O-glucoside	0.071 (0.006)	Luteolin-7-O-glucoside
**16**	Syringetin-3-O-(6-glucosyl)glucoside	0.137 (0.004)	Rutin
**17 + 18 + 19**	Chrysoeriol-7-O-glucoside Isorhamnetin-3-O-glucoside Diosmetin-8-C-(rhamnosyl)-glucoside	0.222 (0.003)	Luteolin-7-O-glucoside
**20**	Quercetin-3-O-(2-rhamnosyl)ketodeoxihexoside	0.412 (0.007)	Rutin
**21 + 22**	Luteolin-8-C-(rhamnosyl)quinovoside Tricin-7-O-glucoside	0.293 (0.004)	Luteolin-7-O-glucoside
**23**	Luteolin-8-C-(2-deoxihexoside)-ketodeoxihexoside	0.72 (0.01)	Rutin
**24**	Luteolin-8-C-(2-rhamnosyl)-ketodeoxihexoside	3.00 (0.02)	Rutin
**25**	Tricin-7-O-glucoside	0.135 (0.009)	Luteolin-7-O-glucoside
**26**	Naringenin	0.140 (0.002)	Acacetin
**27**	Apigenin-7-O-(2-rhamnosyl)ketodeoxihexoside	0.343 (0.006)	Rutin
**28**	Luteolin	0.109 (0.002)	Acacetin
**29**	Apigenin-7-O-(2-rhamnosyl)ketodeoxihexoside	0.54 (0.02)	Rutin
**30**	Luteolin-C-(deoxihexoside)deoxihexoside	0.279 (0.002)	Rutin
**31**	Luteolin-C-(deoxihexoside)hexoside	0.4 (0.03)	Rutin
**32**	Luteolin-C-(deoxihexoside)deoxihexoside	0.26 (0.02)	Rutin
**33**	Kaempferol	0.087 (0.002)	Acacetin
**34**	Luteolin derivate	0.392 (0.043)	Acacetin
**35**	Apigenin	0.290 (0.006)	Acacetin
**36**	Luteolin derivate	0.2 (0.1)	Acacetin
**37**	Luteolin-7-methyl ether	0.137 (0.002)	Acacetin
**38**	Pinocembrin	0.08 (0.01)	Acacetin
**39**	Apigenin-7-O-(4″-p-E-coumaroyl)glucoside	0.226 (0.008)	Luteolin-7-O-glucoside
**40**	Luteolin-8-C-E-propenoic acid	0.52 (0.02)	Acacetin
**41**	Apigenin-7-O-(6″-p-E-coumaroyl)glucoside	0.156 (0.004)	Luteolin-7-O-glucoside
**42**	Apigenin-7-O-(6″-p-Z-coumaroyl)glucoside	0.605 (0.007)	Luteolin-7-O-glucoside
**43**	Apigenin-8-C-propiolic acid	1.97 (0.03)	Luteolin-7-O-glucoside
**44**	Apigenin-7-O-(4″-p-Z-coumaroyl)glucoside	0.501 (0.007)	Luteolin-7-O-glucoside
**45**	Apigenin-7-O-(3″-p-coumaroyl)glucoside	0.234 (0.007)	Luteolin-7-O-glucoside
**46**	Dihydroxyflavone-8-C-propiolic acid	0.387 (0.006)	Acacetin
**47**	Acacetin (5,7-Dihydroxy-4′-methoxyflavone)	0.216 (0.003)	Acacetin
**48**	Genkwanin (4′,5-dihydroxy-7-methoxyflavone)	0.099 (0.005)	Acacetin
**49**	Velutin (5,4′-Dihydroxy-7,3′-dimethoxyflavone)	0.066 (0.001)	Acacetin
	Total flavonoids	15.9 (0.2)	

* Standard deviation in parenthesis (n = 3).

## Data Availability

The original contributions presented in this study are included in the article/Supplementary Material. Further inquiries can be directed to the corresponding author.

## Supplementary Materials

The following supporting information can be downloaded at: https://www.mdpi.com/article/10.3390/ph19040597/s1, Table S1: Cell viability of HepG2 and NHDF cells exposed to increasing concentrations of Liboost^®^. Data are expressed as mean (% of control) ± SD; Table S2: Cell viability of HUVECs after exposure to Liboost^®^; Figure S1: DAD chromatogram at 340 nm of a commercial damiana extract (Liboost^®^). Peak identification of flavonoids according to Table 1; Figure S2: Standard calibration curve for the quantification of phosphodiesterase type 5 (PDE5) based on absorbance measured at 450 nm using the ELISA assay. Data points represent mean values; Figure S3: Standard calibration curve for the quantification of aromatase activity based on fluorescence detection (Ex/Em = 488/527 nm) using the fluorometric aromatase assay kit. Data points represent mean values; Figure S4: Standard calibration curve for nitric oxide (NO) quantification based on nitrite concentration determined by the Griess reaction. Data points represent mean values.

## Abbreviations

The following abbreviations are used in this manuscript:

HPLC-DAD-HRMS	High-Performance Liquid Chromatography—Diode Array Detector—High-Resolution Mass Spectrometry
PDE5	Phosphodiesterase-5
NO	Nitric Oxide
cGMP	cyclic Guanosine Monophosphate
nNOS	Nitric Oxide Synthase
eNOS	Endothelial Nitric Oxide Synthase
GC	Guanylate Cyclase
USD	United States Dollar
CAGR	Compound Annual Growth Rate
US	Unite States
DAD	Diode Array Detector
HPLC-MS	High-Performance Liquid Chromatography—Mass Spectrometry
MW	Molecular Weight
HepG2	Immortalized cell line derived from liver tissue of a patient with hepatocellular carcinoma
NHDF	Normal Human Dermal Fibroblasts
HUVEC	Human Umbilical Vein Endothelial Cells
ANOVA	Analysis of Variance
LC–MS	Liquid Chromatography—Mass Spectrometry
WST-1	Water-Soluble Tetrazolium salt
FBS	Fetal Bovine Serum
EMEM	Eagle’s Minimum Essential Medium
DMEM	Dulbecco’s Modified Eagle Medium
DMSO	Dimethyl Sulfoxide
CO_2_	Carbon Dioxide
SEM	Standard Error of the Mean
HSD	Tukey’s Honestly Significant Difference
