# Supplementary material for: Flavonoid Composition and Molecular Basis of the Potential Sexual-Enhancing Properties of a Turnera diffusa Extract (Liboost®)
Source: Pharmaceuticals (Basel). 2026 Apr 8;19(4):597. doi: 10.3390/ph19040597 (PMC13118355; doi:10.3390/ph19040597)
Supplement: Supplementary file 1 [file pharmaceuticals-19-00597-s001.zip › pharmaceuticals-4221387-supplementary.pdf]

Flavonoid Composition and Molecular Basis of the Potential Sexual-Enhancing Properties of a *Turnera diffusa* Extract (Liboost®)

Iván Benito-Vázquez [1,2], María Inés Morán-Valero [2], Marina Díez-Municio [2] and Adal Mena-García [2,*]

[1] Instituto de Investigación en Ciencias de la Alimentación, CIAL (CSIC-UAM), Nicolás Cabrera 9, 28049 Madrid, Spain; ivan.benito@pharmactive.eu
[2] Pharmactive Biotech Products SLU, Faraday, 7, 28049 Madrid, Spain; ines.moran@pharmactive.eu (M.I.M.-V.); mdiez@pharmactive.eu (M.D.-M.)
* Correspondence: adal.mena@pharmactive.eu; Tel.: +34-656-88-20-49

Supplementary Table S1. Cell viability of HepG2 and NHDF cells exposed to increasing concentrations of Liboost®. Data are expressed as mean (% of control) ± SD.

	**Liboost (mg/mL)**							
	7.8	15.6	31.3	62.5	125	250	500	1000
**Cell type**	**Cell viability (%)**							
HepG2	107.99(4.43)	103.03(5.15)	112.2(5.27)	114.23(6.4)	115.17(5.07)	118.87(7.01)	113.79(5.92)	98.86(3.95)
NHDF	111.19(4.69)	106.07(4.3)	115.52(5.41)	117.61(6.32)	118.58(11.88)	118.69(5.8)	117.16(4.09)	101.78(2.72)

Values represent viability normalized to the untreated control (%). Numbers in parentheses indicate the standard deviation (SD) of three independent replicates (n = 3).

Supplementary Table S2. Cell viability of HUVEC cells after exposure to Liboost®

	**Liboost (µg/mL)**				
	10	40	100	400	1000
**Cell type**	**Cell viability (%)**				
HUVEC	93.6(13.29)	90.38(11)	82.78(15.41)	45.01(5.32)	40.59(5.72)

Values represent viability normalized to the untreated control (%). Numbers in parentheses indicate the standard deviation (SD) of three independent replicates (n = 3).

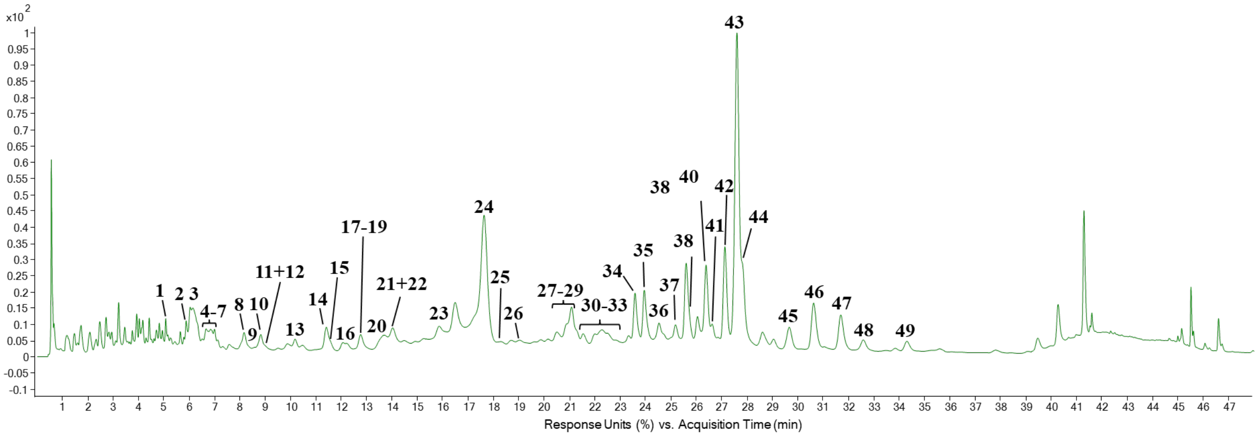

Figure S1. DAD chromatogram at 340 nm of a commercial damiana extract (Liboost®). Peak identification of flavonoids according to Table 1.

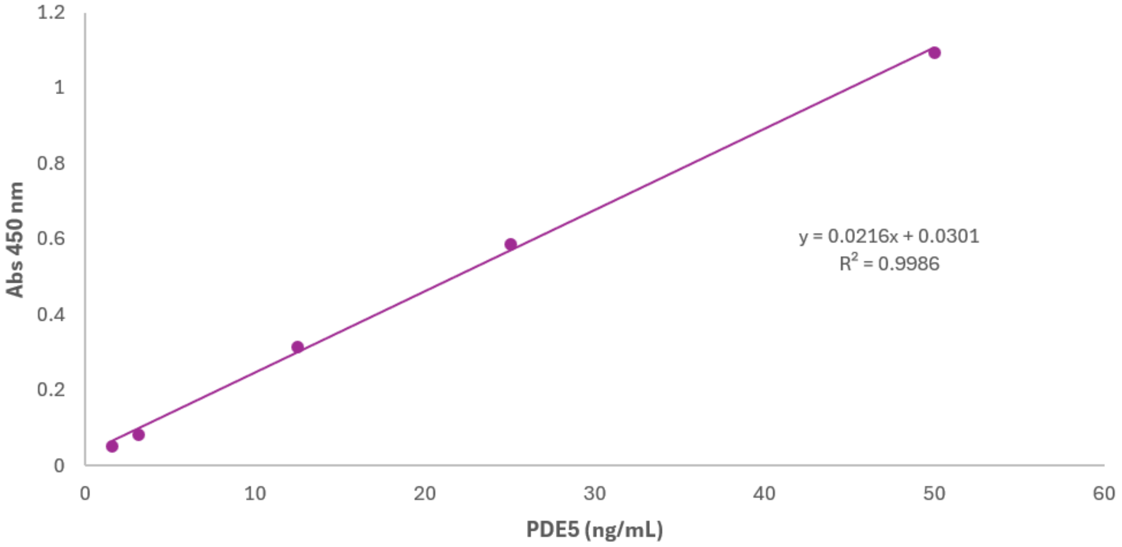

Figure S2. Standard calibration curve for the quantification of phosphodiesterase type 5 (PDE5) based on absorbance measured at 450 nm using the ELISA assay. Data points represent mean values.

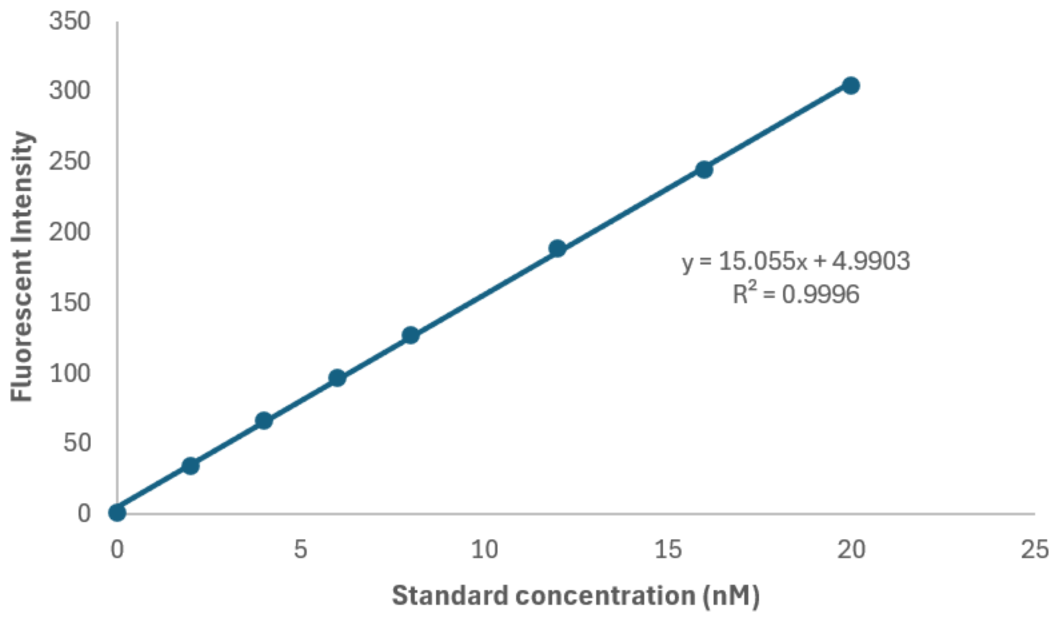

Figure S3. Standard calibration curve for the quantification of aromatase activity based on fluorescence detection (Ex/Em = 488/527 nm) using the fluorometric aromatase assay kit. Data points represent mean values.

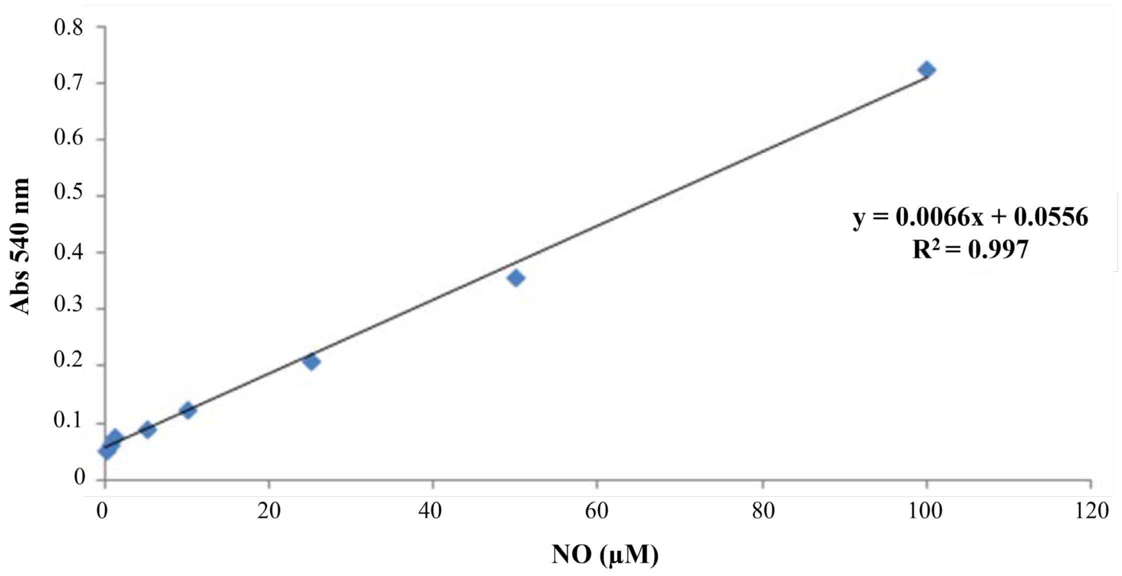

Figure S4. Standard calibration curve for nitric oxide (NO) quantification based on nitrite concentration determined by the Griess reaction. Data points represent mean values.
